# Phylogeny and Selection Pressure of Genus *Chimarrogale* in China Based on Mitochondrial Genomes

**DOI:** 10.3390/ani16101471

**Published:** 2026-05-11

**Authors:** Jiayi Jiang, Xianling Li, Guosheng Jian, Fengjun Li

**Affiliations:** 1 Institute of Ecology, China West Normal University, Shida Road, Nanchong 637009, China; 2 Key Laboratory of Southwest China Wildlife Resources Conservation (Ministry of Education), China West Normal University, Nanchong 637009, China

**Keywords:** *Chimarrogale*, mitochondrial genome, semi-aquatic, selection pressure, phylogeny, molecular evolution

## Abstract

The genus *Chimarrogale* is an ideal group to study the evolutionary mechanisms of semi-aquatic adaptation, but there is a lack of data on its genomic data and molecular mechanisms. Using Illumina sequencing, this study assembled mitogenomes of *C. himalayica* and *C. styani* (newly characterized), alongside *C. leander*, covering all *Chimarrogale* species in China. Results showed that three complete circular mitochondrial genomes were successfully assembled, with full lengths of 17,202–17,218 bp, including the 37 typical genes: 13 protein-coding genes (PCGs), 22 tRNAs, two rRNAs, and a *D-loop* region. There were nine overlapping regions and 14 intergenic spacer regions identified, showing significant AT bias. Relative synonymous codon usage (RSCU) analysis showed that Serine (Ser) was used most frequently. Selection pressure analysis showed that the Ka/Ks ratios of PCGs in 44 Soricidae mitogenomes were less than 1, indicated strong purification selection and functional conservation. Among them, the evolution rate of the *ATP8* gene was the fastest. The phylogenetic analysis using Maximum Likelihood (ML) and Bayesian Inference (BI) methods showed that the three *Chimarrogale* species clustered into a monophyletic clade, which formed a sister group with *Nectogale elegans* within the tribe Nectogalini. This study fills the gap in mitochondrial genome data of semi-aquatic shrews and offers fundamental references for the conservation of shrews.

## Simple Summary

As semi-aquatic shrews, the genus *Chimarrogale* is an excellent model for exploring the evolutionary mechanisms of semi-aquatic adaptation, yet its mitochondrial genomic resources and related molecular studies remain scarce. In this study, we assembled and characterized the complete mitogenomes of three *Chimarrogale* species (*C. himalayica*, *C. styani*, and *C. leander*) using Illumina high-throughput sequencing. The mitogenomes were circular, 17,202–17,218 bp in length, and contained the typical 37 genes (13 protein-coding genes (PCGs), 22 tRNAs, two rRNAs) and a *D-loop* region, with obvious AT bias and purifying selection acting on all PCGs (Ka/Ks < 1). Phylogenetic analyses revealed that *Chimarrogale* form a monophyletic clade, which is a sister group to *Nectogale elegans* within the tribe Nectogalini. This work enriched the mitochondrial genomic data of semi-aquatic shrews and provides crucial genetic resources for investigating the molecular mechanisms of semi-aquatic adaptation and the phylogenetic relationships of shrews.

## 1. Introduction

A total of 70 species in 12 genera of the family Soricidae are distributed in China, occupying diverse habitats and broad environmental gradients, thus rendering it an exemplary model system for investigating adaptive evolution [1,2]. Water shrews are a group of small semi-aquatic mammals [3,4,5]. Among them, species of *Chimarrogale* represent an example of semi-aquatic adaptation, with a broad distribution spanning northern India, Nepal, Myanmar, Japan, and China [2,6,7]. Within China, the genus is primarily documented in the provinces of Yunnan, Sichuan, and Zhejiang, and the genus comprises three species, including *Chimarrogale himalayica*, *Chimarrogale styani*, and *Chimarrogale leander* [2,8].

Semi-aquatic shrews recognized in China mostly belong to the tribe Nectogalini within the family Soricidae [4,8,9,10,11]. Phylogenetic analysis based on the mitochondrial *CYTB* gene has shown that there are six genera of the tribe Nectogalini, including *Chodsigoa*, *Episoriculus*, *Soriculus*, *Neomys*, *Nectogale*, and *Chimarrogale* [12,13]. Research on the genus *Chimarrogale* has focused predominantly on morphological descriptions, with molecular insights largely confined to short mitochondrial fragments such as *CYTB* and *ATP6* [7,14]. A multi-locus analysis corroborated a European origin for the tribe Nectogalini, which includes *Chimarrogale*, and has shown that this tribe subsequently dispersed across Central Asia, indicating that aquatic specializations evolved through multiple independent events [15]. A previous study inferred up to ten cryptic lineages within the genus, recovered *C. himalayica* as paraphyletic, and proposed the elevation of *C. leander* to full specific status by analyzing the *CYTB* fragment [16]. The study also postulated that the tribe Nectogalini colonized Asia from Europe via a low-latitude, trans-Central Asian route [16]. Although earlier studies have situated the Nectogalini’s aquatic radiation within a macro-evolutionary timeframe congruent with Tibetan Plateau uplift [17], the genomic architecture underpinning this adaptive transition awaits comprehensive elucidation [8,15]. And, the scarcity of genetic data has precluded robust resolution of interspecific relationships.

The simple structure, maternal inheritance, and relatively rapid evolutionary rate of complete mitochondrial genomes make them valuable markers for high-resolution phylogenetic inference, especially at shallow evolutionary scales (e.g., among closely related species or populations) [18,19]. For instance, in sequencing the complete mitochondrial genome of *Episoriculus leucops*, research has unequivocally placed *E. leucops* within the subfamily Soricinae and identified *Episoriculus caudatus* as its closest relative species [20]. The mitochondrial genome has also been used as a barcode in Eutherian mammals [21]. Key metrics, such as the ratio of nonsynonymous substitution rate (Ka) to synonymous substitution rate (Ks), help identify selective pressures, with Ka/Ks < 1 indicating purifying selection and Ka/Ks > 1 suggesting positive selection [22]. Adaptive changes in protein-coding genes (PCGs), such as *COX1* and *ND5*, have been observed in high-altitude mammals and deep-sea fish, reflecting metabolic demands [23]. Structural and regulatory changes, alongside genetic diversity analyses, provide insights into population history and environmental adaptations [24]. Furthermore, comparative mitochondrial genomes can reveal lineage-specific evolutionary rates [25,26].

Mitochondrial DNA (mtDNA) evolves at a higher rate than nuclear DNA due to limited repair mechanisms and oxidative stress exposure, making it a valuable tool for studying adaptive evolution [19,25]. However, as a single, maternally inherited linkage group, mtDNA provides only a partial perspective on evolutionary history and may not fully reflect complex processes such as hybridization or male-mediated gene flow [27,28]. Currently, among the three *Chimarrogale* species distributed in China, only *C. leander* has a publicly available complete mitochondrial genome (NCBI accession: NC_063622.1) [29]. The lack of mitogenomic data for the remaining two species hampers robust inference of interspecific genetic architecture, divergence relationships, and mitogenomic structural variation.

To address this data gap and resolve key phylogenetic uncertainties, this study sequenced, assembled, and annotated the complete mitochondrial genomes of all three *Chimarrogale* species distributed in China. Using Illumina sequencing technology, this study conducted comparative analyses of genome size, nucleotide composition, AT- and GC-skew, codon usage bias, and selection pressures across the 13 PCGs among the three species. Furthermore, we used all available mitogenomes of Soricidae in China to assess selection pressures on the PCGs across a broader phylogenetic context of shrews. Based on the dataset of PCGs, we reconstructed robust Bayesian Inference (BI) and Maximum Likelihood (ML) phylogenies of shrews in China, thereby providing the essential genetic framework required to clarify the systematic and evolutionary history of semi-aquatic shrews.

## 2. Materials and Methods

### 2.1. Samples and Mitochondrial Genome Sequencing

In this study, muscle tissue samples from three *Chimarrogale* species were selected: *Chimarrogale himalayica* from Mabian County, Leshan City, Sichuan Province, China, *Chimarrogale leander* from Taojiang County, Huaihua City, Hunan province, China, and *Chimarrogale styani* from Lanping County, Nujiang City, Yunnan Province, China. After morphological identification, samples were placed in 95% ethanol and stored at −80 °C for use. All procedures were approved by the Ethics Committee of China West Normal University (approval No. CWNU2023D003). A Blood and Tissue Kit (Novogene Co., Ltd., Beijing, China) was used to extract the total genomic DNA of samples. The purity and concentration of DNA were detected using a Nanodrop 1000 microspectrophotometer (Thermo Fisher Scientific, Wilmington, DE, USA) and a Qubit fluorometer (Thermo Fisher Scientific, Wilmington, DE, USA), respectively, to ensure that the OD260/OD280 ratio was between 1.8 and 2.0. The mitochondrial *CYTB* sequences were firstly amplified using the primers H15915_hk3/L14724_hk3 [15]. The annealing temperature was 49 °C and the PCR cycle was 35 times. Molecular identification on the *CYTB* sequence of the samples was performed through NCBI BLAST (https://blast.ncbi.nlm.nih.gov/Blast.cgi (accessed on 13 May 2024)) to confirm that its similarity was greater than 98% before proceeding with next-generation sequencing. Next-generation sequencing was performed on the Illumina platform, and the sequencing depth was 10 Gb–40 Gb. Primary quality control was carried out by Berry Biotechnology Co., Ltd., Beijing, China, with the following criteria: firstly, paired-end reads were discarded if the number of ambiguous bases (N) in a single-end read exceeded 3 and the number of bases with a Phred quality score 5 accounted for >20% of the total read length; secondly, adapter sequences were trimmed when the matching length between the read and the adapter exceeded 8 bp. After passing the above filters, high-quality, clean data were used for subsequent mitochondrial genome assembly and analysis.

### 2.2. Assembly and Annotation of the Mitochondrial Genome

The complete mitochondrial genome was assembled using MitoZ v3.6 [30], which is a dedicated tool for mitogenome reconstruction that integrates assembly, annotation, and visualization. MitoZ v3.6 employed a hybrid assembly strategy. First, it used Megahit, a built-in assembler module within MitoZ v3.6, to construct initial contigs from high-quality clean reads. Then, identification of mitochondrial contigs via homology was searched against a mitochondrial reference database. Finally, the circular mitochondrial genome was generated through iterative extension. The initial assembly command was set as follows: length of the inserted segment was 300 bp, length of the sequencing read was 150 bp, the kmers were 21, 39, 59, 79, 99, and 119, and the designated evolutionary branch was Chordata. When screening mitochondrial candidate sequences for the initial assembly of *Chimarrogale styani*, no eligible sequences were found with a data depth of 10 Gb, which was insufficient to assemble the mitochondrial genome of *C. styani*. Therefore, we increased the sequencing depth to 40 Gb, and then MitoZ v3.6successfully yielded the mitochondrial genome for *C. styani*. The same assembly parameters were used for all three *Chimarrogale* species. The annotation module of MitoZ v3.6 software was used to annotate the mitochondrial genome, with the designated genetic code set to 2. The visualization module of MitoZ was used to generate a circular map of mitochondrial genome diversity. Nucmer tool v3.1 [31] was used for collinearity alignment to verify the integrity of the assembled sequence. Using the existing *Chimarrogale leander* (NC_063622.1) as the reference sequence, the *C. leander* mitochondrial genome sequence assembled in this study was aligned to verify integrity. Then, the Mummerplot v3.5 [31] tool was used to draw a collinearity comparison chart.

### 2.3. Analysis of Mitochondrial Genome Characteristics

Bioawk v1.0 (https://github.com/lh3/bioawk (accessed on 17 December 2025)) was used to count the number of A, T, C, and G bases and to determine the skew calculation according to AT-skew = (A − T)/(A + T), GC-skew = (G − C)/(G + C) (avoid dividing by 0; when A + T = 0, AT skew = 0, and G + C = 0, GC skew = 0). We calculated the value of relative synonymous codon usage frequency (RSCU) according to RSCU = (codon usage/total usage of this amino acid) × number of synonymous codons. Vmatch v2.3.0 (http://vmatch.de/ (accessed on 22 December 2025)) software was employed to identify interspersed repetitive sequences; the minimum repeat length was set to 20 bp, and 3 mismatches were allowed. Four categories of repetitive sequences were targeted for identification, namely, Forward (F), Palindrome (P), Complementary (C), and Reverse (R). The statistical analysis of mitochondrial characteristics was uniformly conducted using the H chain.

### 2.4. Selection Pressure Analysis

We searched the NCBI database and retrieved complete mitochondrial genomes for shrews in China up to 23 December 2025 (Table 1). The KaKs_Calculator v2.1 [32] tool was employed to calculate the nonsynonymous substitution rate (Ka), synonymous substitution rate (Ks), and Ka/Ks ratio for PCGs of these mitochondrial genomes available in NCBI (Table 1), as well as the three mitochondrial genomes assembled in this study. Two groups of PCG evolutionary selection pressures were analyzed, including shrews in China (44 in total) and *Chimarrogale* shrews in China (3 in total).

### 2.5. Phylogenetic Analysis

Using the available mitochondrial genomes of Chinese shrews and the three *Chimarrogale* species, with *Uropsilus andersoni* (NC_041144.1) and *Uropsilus soricipes* (NC_023244.1) designated as outgroups, we selected 13 PCGs as molecular markers and aligned them using MAFFT v7.526 [33]. The phylogenetic tree was constructed using the Maximum Likelihood (ML) and Bayesian Inference (BI) methods. For ML tree construction, IQ-TREE v3.0.1 [34] was used with 1000 ultrafast bootstrap replicates. For BI tree construction, MrBayes v3.2.7 [35] software was employed, which set four simultaneous Markov Chain Monte Carlo (MCMC) algorithms, ran for 2 million generations, and discarded the first 25% of the data as burn-in. The phylogenetic tree was visualized using the online tool iTOL v7 [36]. R v4.3.1 (ggplot2 package) [37] was used for visual analysis.

## 3. Results

### 3.1. Mitochondrial Gene Organization and Features

**Figure 1:**
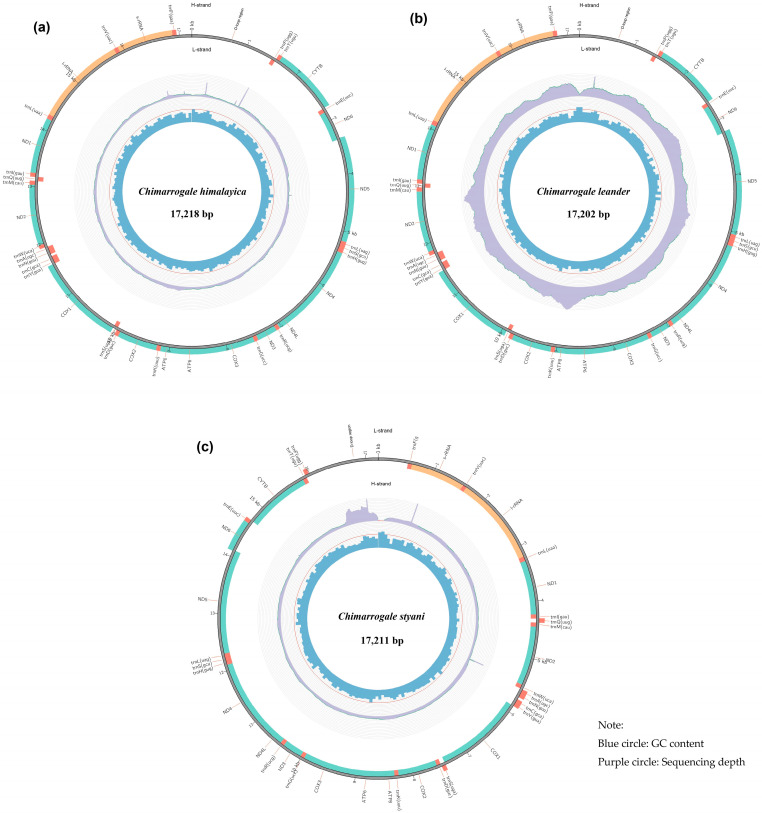
Circular map of the mitochondrial genome of three *Chimarrogale* species. (**a**) *Chimarrogale himalayica*; (**b**) *Chimarrogale leander*; (**c**) *Chimarrogale styani*.

Up to 23 December 2025, we searched the NCBI database and retrieved complete mitochondrial genomes for 41 shrews in China (Table 1). In this study, the mitochondrial genomes of three species of *Chimarrogale* were sequenced and assembled: *Chimarrogale himalayica* (CH), *Chimarrogale leander* (CL), and *Chimarrogale styani* (CS). After quality control of the next-generation sequencing data, the valid data were as follows: CH: 9.9 Gb (28,554,847 reads); CL: 8.3 Gb (23,946,430 reads); and CS: 41.0 Gb (116,853,879 reads). The complete length of the mitochondrial genomes of the three *Chimarrogale* species was determined as 17,218 bp (CH), 17,202 bp (CL), and 17,211 bp (CS), respectively (Figure 1). All three species possessed a closed circular double-stranded mitochondrial genome, which contained a total of 37 genes. These genes included 13 PCGs (*ND1*, *ND2*, *COX1*, *COX2*, *ATP8*, *ATP6*, *COX3*, *ND3*, *ND4L*, *ND4*, *ND5*, *ND6*, *CYTB*), 22 tRNAs, two rRNAs, and one *D-loop* region (Tables S1–S3). Notably, one protein-coding gene (*ND6*) and eight transfer RNA (tRNA) genes were located on the light strand (L-strand), while the remaining 28 genes were distributed on the heavy strand (H-strand) (Figure 1).

Analyses of the mitochondrial genomes of the three species revealed that all of them contained nine overlapping regions and 14 intergenic spacer regions (Tables S1–S3). The total length of the overlapping regions was identical at 78 bp across the three species, whereas the total length of the intergenic spacer regions was 72 bp (CH), 73 bp (CL), and 70 bp (CS), respectively. The largest intergenic spacer region, spanning 42–43 bp in the three species, was located between *tRNA-Asn* and *tRNA-Cys*. Furthermore, the maximum length of the gene overlapping regions was 43 bp in all three species, which is located between *ATP6* and *ATP8*.

The nucleotide composition of mitogenomes of the three *Chimarrogale* species was as follows: A content ranged from 31.26% to 31.59%, T content from 31.22% to 33.48%, C content from 12.60% to 12.81%, and G content from 22.33% to 22.89% (Table S4). The three species exhibited relatively high A + T contents (64.48–65.07%) and low G + C contents (34.93–35.70%), indicating a distinct bias toward AT base pairs. In addition, three *Chimarrogale* species had negative AT-skew values and positive GC-skew values, which indicated that T content was higher than A content and G content was higher than C content in these two species (Table S4).

**Figure 2:**
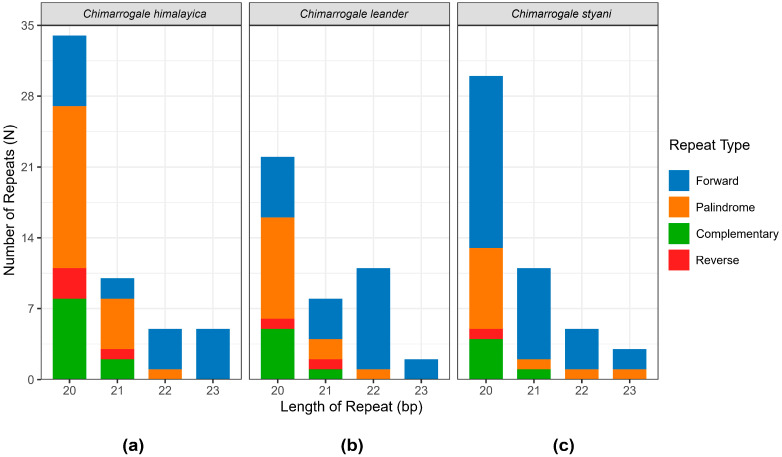
Statistical plot of scattered repeat sequences in the mitochondrial genome of three *Chimarrogale* species. (**a**) *Chimarrogale himalayica*; (**b**) *Chimarrogale leander*; (**c**) *Chimarrogale styani*.

Among the mitogenomes of the three *Chimarrogale* species, the number of repeat sequences with a fragment length of 20 bp was the highest, and the number of repeat sequences with a fragment length of 20 bp in CH was higher than in CL and CS (Figure 2). In addition, the second most abundant repetitive sequence length was 21 bp in both CH and CS, whereas it was 22 bp in CL. The *D-loop* regions of the three *Chimarrogale* species were all located between *tRNA-Pro* and *tRNA-Phe*, with lengths of 1750 bp (CH), 1739 bp (CL), and 1741 bp (CS), respectively (Tables S1–S3). In all three species, the *D-loop* region exhibited significant AT enrichment characteristics.

### 3.2. Protein-Coding Genes and Codon Usage

**Figure 3:**
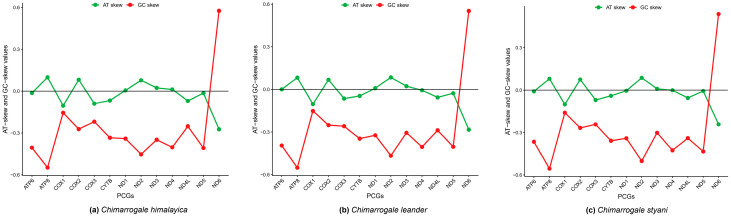
AT-skew and GC-skew values in PCGs of the mitochondrial genome of three *Chimarrogale* species. (**a**) *Chimarrogale himalayica*; (**b**) *Chimarrogale leander*; (**c**) *Chimarrogale styani*.

The complete sequence length of 13 PCGs in the mitogenomes of CH and CL was 11,421 bp, while in CS it was 11,424 bp (*ND5* is 3 bp longer). The PCGs accounted for 66.33% (CH), 66.39% (CL), and 66.38% (CS) of the total mitogenome length, respectively. Among these three species, 12 PCGs were located on the heavy chain (H-strand), while only the ND6 gene was located on the light chain (L-strand). The AT-skew and GC-skew values of most PCGs were negative, indicating high content of T and C (Figure 3). Notably, the GC-skew values were lower than the AT-skew values, and the *ND6* gene showed the most significant fluctuation in AT/GC-skew values across all three species (Figure 3). Ten PCGs of three species used ATG as the starting codon, while only *ND2*, *ND3*, and *ND5* genes used ATA as the starting codon. There were differences in the termination codons of PCGs: the *ND4* gene used a single T as the termination codon, the *COX3* gene used TA as the termination codon, and *CYTB* used AGA as the termination codon. In CL, eight PCGs (*COX1*, *COX2*, *ATP8*, *ATP6*, *ND3*, *ND4L*, *ND5*, *ND6*) used TAA as the termination codon, while in CH and CS all seven remaining PCGs used TAA as the termination codon, except for the *COX1* gene, which used TAG (Tables S1–S3).

**Figure 4:**
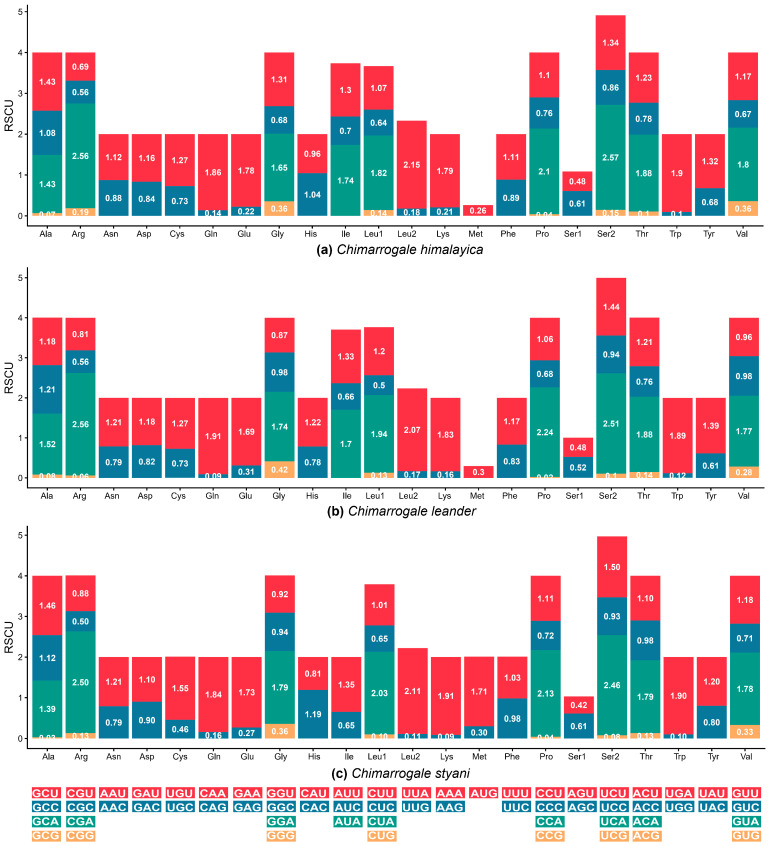
Relative synonymous codon usage (RSCU) of the mitochondrial genome of three *Chimarrogale* species. (**a**) *Chimarrogale himalayica*; (**b**) *Chimarrogale leander*; (**c**) *Chimarrogale styani*.

The mitogenomes of both CH and CL encoded 3807 amino acids, while that of CS encoded 3808 amino acids. Among them, Serine (Ser) exhibited the highest usage frequency. The most frequently used codon for CH was UCA (Ser2), whereas the most frequently used codon for CL and CS was CGA (Arg) (Figure 4).

### 3.3. Collinearity Analysis of Mitochondrial Genomes

**Figure 5:**
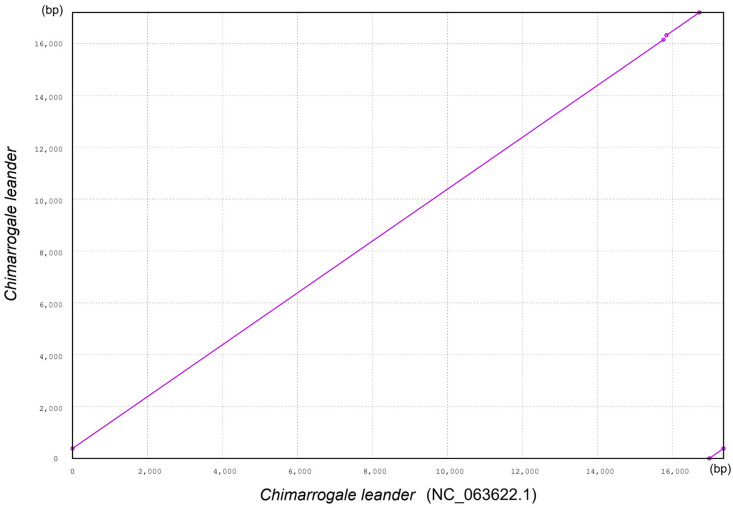
Collinearity alignment between the reference mitogenome of *Chimarrogale leander* (NC_063622.1) and the assembled mitogenome of *Chimarrogale leander* in this study.

Using the reported CL mitogenome (NC_063622.1) as a reference sequence, collinearity alignment was performed on the assembled CL mitogenome sequence in this study to verify integrity. The results showed that these two CL mitogenome sequences exhibited high collinearity (Figure 5). In the collinearity comparison graph, there was a high completion linear positive correlation between the reference sequence and the assembly sequence, indicating that the gene arrangement order and fragment continuity of the CL mitogenome assembled in this study were highly consistent with those of the reference sequence. The core matching region covered the entire sequence length, with high consistency in the aligned intervals. Only short-fragment matches were detected in the terminal regions, and there was no significant large fragment rearrangement, deletion, or insertion. This result confirmed the accuracy and completeness of the mitogenome assembly sequence in this study.

### 3.4. Selective Pressure Analysis

**Figure 6:**
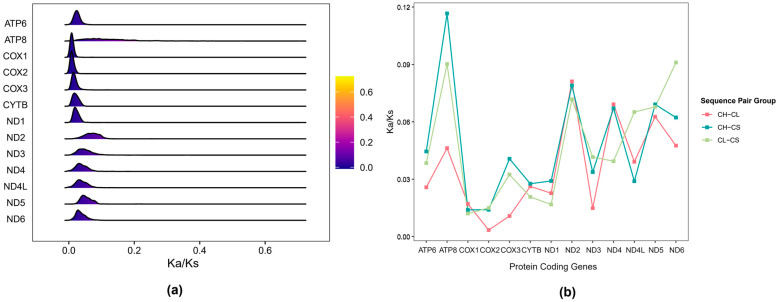
Ka/Ks values for each PCG in pairs of mitochondrial genomes. (**a**) Shrews in China (44 in total); (**b**) shrews in the genus *Chimarrogale* in China.

The ratio of Ka/Ks was used to evaluate the selection pressure on 13 PCGs in 44 shrews (Figure 6a) and three *Chimarrogale* species (Figure 6b). The results showed that the Ka/Ks ratios of all PCGs were less than 1, indicating that these genes were subjected to purifying selection and their functional conservation was strictly maintained during the evolutionary process (Figure 6). The Ka/Ks ratios of the 13 PCGs exhibited significant variations across the Soricidae species, ranging from 0.03 to 0.27 (Figure 6a). The Ka/Ks ratio of the *ATP8* gene was the highest, indicating that this gene has a relatively faster evolutionary rate and relatively relaxed functional constraints. The Ka/Ks ratio of *COX1* and *COX2* genes was the lowest, indicating that these two genes bear the stronger purification selection pressure and have the slower evolutionary rate (Figure 6a). The Ka/Ks ratios of three *Chimarrogale* species were low, and the differences between genes were flat (Figure 6b). The Ka/Ks ratios of *COX1*, *COX2*, and *CYTB* genes remained low, while the *ATP8* gene remained the highest ratio. This result indicated that the PCGs of *Chimarrogale* are subjected to strong purification selection and have stronger functional conservation. Meanwhile, the relatively fast evolution of the *ATP8* gene might represent a shared characteristic of *Chimarrogale* species in their adaptation to the aquatic environment, which provides a certain degree of evolutionary flexibility for their energy metabolism efficiency.

### 3.5. Phylogenetic Relationships

**Figure 7:**
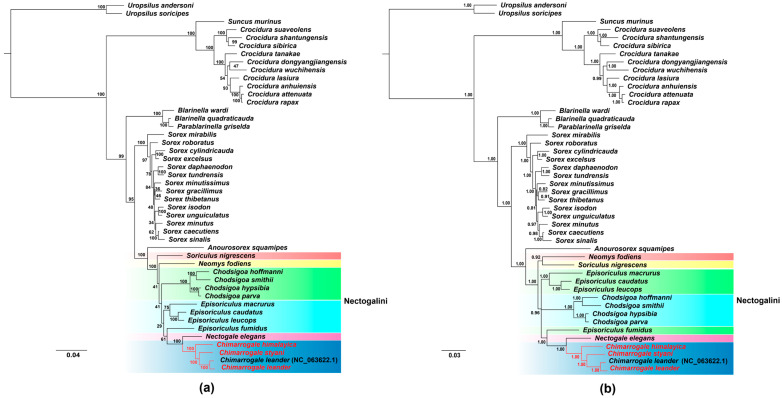
The phylogenetic tree reconstructed from 13 PCGs of shrews in China. (**a**) Maximum Likelihood (ML) tree. (**b**) Bayesian Inference (BI) tree.

Phylogenetic analyses were performed based on the concatenated sequence of 13 mitochondrial PCGs using the Maximum Likelihood (ML, Figure 7a) and Bayesian Inference (BI, Figure 7b) methods. The results showed that the phylogenetic trees constructed using the two methods exhibited overall congruent topological structures, with high support values for the core clades. However, there were slight differences in the clustering relationship and support strength of some secondary branches, which verified the reliability of the phylogenetic relationship (Figure 7).

The ML tree supported the core branch with high bootstrap values, with the bootstrap value (BS) of most key nodes ≥ 95 (Figure 7a). The tree topology was clearly divided into six major core clades, corresponding to the known primary taxa of the family Soricidae. Both *Neomys fodiens* and *Nectogale elegans* are representative species exhibiting semi-aquatic adaptations. Among them, three *Chimarrogale* species and *N. elegans* clustered into one branch (BS = 100). This clade constituted a fully independent lineage together, but *N. fodiens* was positioned at the base of the tribe Nectogalini, which clarified its status as an early differentiation taxon. In addition, the species within the genus *Sorex* showed tight clustering, with generally high support values for the secondary clades (BS = 62 to 100), reflecting the close genetic relationships among these species.

The BI tree, which evaluates branch reliability based on posterior probability, showed core nodes with posterior probabilities (PP) of ≥0.95 (Figure 7b). Compared to the ML tree, the BI tree exhibited higher resolution for secondary branches. For instance, differences were observed between the two methods in the clustering order of certain species within the genus *Sorex* (e.g., *S. minutissimus* and *S. unguiculatus*), with the posterior probability of the corresponding node in the BI tree (PP = 0.97) being higher than the bootstrap value in the ML tree (BS = 34). In the tribe Nectogalini, the BI tree also supported the clustering of the three *Chimarrogale* species into one clade (PP = 1.00) and their sister group relationship with *Nectogale elegans* (PP = 1.00). Meanwhile, *Neomys fodiens* was confirmed as the relatively basal taxon (PP = 1.00) in the tribe Nectogalini, further verifying the stability of this divergence pattern. In summary, the phylogenetic trees constructed by both independent methods clearly clarified the core divergence framework of the Soricidae, especially the independent differentiation relationship between the ‘*Chimarrogale* and *Nectogale*’ branch in the tribe Nectogalini.

## 4. Discussion

In this study, the complete mitochondrial genomes of all species of the genus *Chimarrogale* distributed in China were successfully characterized, including *Chimarrogale himalayica* (CH), *Chimarrogale leander* (CL), and *Chimarrogale styani* (CS). In this study, the mitochondrial genomes of CH and CS were identified and systematically analyzed for the first time, whereas the CL genome had been sequenced previously [29]. We further improved sequencing depth and reassembly validation for the CL genome. This study provides crucial and reliable molecular data support for further exploring the molecular evolution patterns of water shrews and their unique semi-aquatic adaptability genetic mechanisms.

The mitogenomes of the water shrews were all closed circular structures. They contained 37 typical genes and one *D-loop* region, which is consistent with the typical characteristics of vertebrate mitochondrial genomes [38,39]. In this study, the proportion of 20 bp repeat sequences was the highest among the three species, and there were species-specific differences in the distribution of repeat sequence lengths between CH, CL, and CS. The abundant repetitive sequences in the mitochondrial genomes of the three *Chimarrogale* species played an important role in their evolutionary processes. A study of deep-sea snails has shown that transposons can affect genome size, promote genome rearrangement, influence gene expression levels, and alter gene regulatory networks [40]. Previous studies have shown that repetitive sequences in the mitogenome can participate in species evolution by affecting gene expression and promoting genome rearrangement [39,41]. The abundant repetitive sequences in the mitogenome of water shrews may provide genetic plasticity for their semi-aquatic adaptive evolution. In addition, all three species have nine overlapping regions and 14 gene gap regions. This gap region served as the starting point for light chain replication, and its conservation reflected the stability of the mitochondrial DNA replication mechanism [42]. The gene overlap between *ATP6* and *ATP8* helped to improve genome compactness and transcription efficiency, as shown for fish of the family Gerreidae [43]. This may be one of the molecular strategies for water shrews to adapt to energy metabolism requirements in semi-aquatic environments.

The nucleotide composition analysis showed the A+T base preference of the mitogenomes of the three species, which is consistent with the characteristics of most vertebrate species and is related to the replication and transcription mechanisms of mitogenomes [41,44]. In PCGs, the AT and GC bias values of most genes were negative, and the GC bias value was always greater than the AT bias value. Among them, the bias value of the *ND6* gene significantly fluctuated, which is related to the function of the key subunit of mitochondrial complex I encoded by the *ND6* gene [45]. This gene is involved in electron transfer and proton transport, and its respiratory-metabolism-related selection pressure is significantly different from other genes.

Codon usage analysis showed that most PCGs start with ATG, while *ND2*, *ND3*, and *ND5* start with ATA. This result differed from the start codon usage patterns of some teleosts [46], which reflect the unique translation start regulation mechanism of mammals. The use of stop codons exhibits species specificity, including TAA, TAG, and incomplete stop codon (TA and single T). These incomplete stop codons can be modified through post-transcriptional polyadenylation to form complete stop codons, and the species-specific distribution of complete stop codons highlights the dynamic evolutionary characteristics of mitogenomes [47]. In terms of amino acid usage, Serine (Ser) was the most frequently used amino acid in this study. Serine serves as a precursor for the biosynthesis of other biomolecules, playing an indispensable role in maintaining the structural integrity and functional efficiency of mitochondrial proteins [48]. This preference may be an adaptive strategy for improving translation efficiency under natural selection.

The high collinearity between the CL assembly sequence in this study and the reference sequence not only validated the reliability of the assembly results but also reflected the structural conservation of mitogenomes in shrews. The almost complete collinearity of the two sequences indicates that the mitogenome of this species had undergone strong purification selection during evolution, and its gene structure had not undergone adaptive changes. The mitogenome, as a closed-loop molecule inherited from the maternal lineage, is typically subject to strict functional constraints in terms of gene sequence and fragment continuity and is crucial for maintaining transcription and translation efficiency [49]. This is related to the high metabolic demand of shrews, which relies on the stability of mitochondrial function.

The selection pressure analysis showed that Ka/Ks ratios of PCGs in shrews and species in the genus *Chimarrogale* in China were all less than 1. The results indicated that these genes are generally subjected to purification selection and functional conservation is strictly preserved in evolution, as demonstrated in various mammalian species [50]. Mitochondrial PCGs encode key aerobic respiratory chain proteins, such as cytochrome c oxidase and ATP synthase, which directly participate in energy production and are the basis for shrews to maintain high metabolic rates, constant body temperature, and an active lifestyle [51]. These genes requires high conservation to ensure functional stability, and any amino acid changes may disrupt protein function and affect energy supply [19]. Therefore, purification selection effectively inhibits mutation diffusion and maintains the stability of core metabolic functions by eliminating individuals carrying harmful nonsynonymous mutations [19,52]. As typical housekeeping genes, the conservation of mitochondrial PCGs is the core guarantee for the basic survival ability of shrews [53]. From an evolutionary perspective, strong purification selection is an important support for shrews to adapt to their environment [52]. As a small mammal, shrews face challenges such as fast energy consumption and high environmental pressure, and the stability of mitochondrial gene function directly determines their ability to cope with the environment. This selection mode avoids the adaptive decline caused by variations in core metabolic genes, providing molecular guarantees for its stable reproduction in diverse ecological niches [18].

The Ka/Ks ratio of the *ATP8* gene was the highest, indicating that its evolutionary rate is relatively fast and its functional constraints are relatively relaxed. Similar data have been obtained for other vertebrates, including catfish of the genus *Glyptothorax* [54]. The relatively rapid evolution of the *ATP8* gene may be a common feature maintained in their adaptation to aquatic environments, providing evolutionary flexibility for energy metabolism efficiency. The Ka/Ks ratio of *COX1* and *COX2* genes was the lowest, indicating that they undergo strong purification selection [55]. Their slow evolution rate is closely related to their core functions in the cytochrome c oxidase complex. These genes are crucial for energy metabolism and functional mutations, which may lead to decreased adaptability [49].

The phylogenetic analysis showed that the three species clustered into one branch (BS = 100, PP = 1.00) and then formed a sister group with *Nectogale elegans* using both ML and BI methods. *Neomys fodiens* and *Soriculus nigrescens* formed an earlier-diverging lineage within the tribe Nectogalini. This result showed the phylogenetic status of the genus *Chimarrogale*, confirmed the independent differentiation relationship between the ‘*Chimarrogale* and *Nectogale*’ branch and *N. fodiens*, providing molecular evidence for the taxonomy of the semi-aquatic shrew [15,56]. The system trees constructed using the two methods have slight differences in the secondary branches, such as the clustering order of some species in the genus *Sorex*, but the BI tree has higher resolution for some nodes. This is attributed to the BI method’s integration of prior probabilities and MCMC-based parameter uncertainty analysis, which can resolve ambiguous phylogenetic relationships at poorly supported nodes compared to ML, which focuses on likelihood optimization alone [57].

This study provided a comprehensive analysis of the mitochondrial genome characteristics and phylogenetic relationships of species in the genus *Chimarrogale* in China. Future research may expand the sampling range to include more species and closely related groups of water shrews, such as *C. phaeura*, *C. platycephalus*, and *C. varennei*, further improving the phylogenetic framework of semi-aquatic shrews.

## 5. Conclusions

This study characterized the complete mitochondrial genomes of all three Chinese *Chimarrogale* species (*C. himalayica*, *C. leander*, *C. styani*). Three mitogenomes were conserved with a closed circular structure and contained 37 typical genes and one *D-loop* region. They had significant AT bias, consistent with vertebrate traits. Selection pressure analysis showed that PCGs undergo purifying selection, implying the stability of mitochondrial function’s role in the environmental adaptability of shrews. ML/BI phylogenies confirmed *Chimarrogale* monophyly, forming a sister group with *Nectogale elegans* of the tribe Nectogalini. The results of the present study have provided basic data and references for the genus *Chimarrogale*.

## Figures and Tables

**Table 1:** GenBank accession numbers of mitogenome sequences of Soricidae species in China used in this study.

Genus	English Name	Species Name	NCBI ID
*Anourosorex*	Chinese Mole Shrew	*Anourosorex squamipes*	NC_024563.1
*Blarinella*	Asiatic Short-Tailed Shrew	*Blarinella quadraticauda*	NC_023950.1
*Blarinella*	Burmese Short-Tailed Shrew	*Blarinella wardi*	NC_041145.1
*Chimarrogale*	Himalayan Water Shrew	*Chimarrogale leander*	NC_063622.1
*Chodsigoa*	Hoffmann’s Long-Tailed Shrew	*Chodsigoa hoffmanni*	MK940327.1
*Chodsigoa*	De Winton’s Shrew	*Chodsigoa hypsibia*	NC_060870.1
*Chodsigoa*	Pygmy Brown-Toothed Shrew	*Chodsigoa parva*	NC_053858.1
*Chodsigoa*	Smith’s Shrew	*Chodsigoa smithii*	MN038168.1
*Crocidura*	Anhui White-Toothed Shrew	*Crocidura anhuiensis*	NC_088563.1
*Crocidura*	Asian Gray White-Toothed Shrew	*Crocidura attenuata*	NC_026204.2
*Crocidura*	Dongyangjiang White-Toothed Shrew	*Crocidura dongyangjiangensis*	NC_056167.1
*Crocidura*	Ussuri White-Toothed Shrew	*Crocidura lasiura*	NC_029329.1
*Crocidura*	Chinese White-Toothed Shrew	*Crocidura rapax*	OR992092.1
*Crocidura*	Shantung White-Toothed Shrew	*Crocidura shantungensis*	NC_021398.1
*Crocidura*	Siberian Shrew	*Crocidura sibirica*	MH349094.1
*Crocidura*	Lesser White-Toothed Shrew	*Crocidura suaveolens*	MW815431.1
*Crocidura*	Taiwanese Gray White-Toothed Shrew	*Crocidura tanakae*	NC_046831.1
*Crocidura*	Hainan White-Toothed Shrew	*Crocidura wuchihensis*	NC_079638.1
*Episoriculus*	Hodgsons’s Brown-Toothed Shrew	*Episoriculus caudatus*	NC_026131.1
*Episoriculus*	Taiwanese Brown-Toothed Shrew	*Episoriculus fumidus*	NC_003040.1
*Episoriculus*	Long-Tailed Brown-Toothed Shrew	*Episoriculus leucops*	NC_056333.1
*Episoriculus*	Long-Tailed Mountain Shrew	*Episoriculus macrurus*	NC_029840.1
*Nectogale*	Elegant Water Shrew	*Nectogale elegans*	NC_023351.1
*Neomys*	Eurasian Water Shrew	*Neomys fodiens*	NC_025559.1
*Parablarinella*	Gray Short-Tailed Shrew	*Parablarinella griselda*	MN873563.1
*Sorex*	Laxmann’s Shrew	*Sorex caecutiens*	MF374796.1
*Sorex*	Stripe-Backed Shrew	*Sorex cylindricauda*	NC_025278.1
*Sorex*	Siberian Large-Toothed Shrew	*Sorex daphaenodon*	NC_044107.1
*Sorex*	Chinese Highland Shrew	*Sorex excelsus*	KY676803.1
*Sorex*	Slender Shrew	*Sorex gracillimus*	NC_037859.1
*Sorex*	Taiga Shrew	*Sorex isodon*	MG983792.1
*Sorex*	Eurasian Least Shrew	*Sorex minutissimus*	NC_042196.1
*Sorex*	Eurasian Pygmy Shrew	*Sorex minutus*	MN122904.1
*Sorex*	Ussuri Shrew	*Sorex mirabilis*	MF438265.1
*Sorex*	Flat-Skulled Shrew	*Sorex roboratus*	NC_034808.1
*Sorex*	Chinese Shrew	*Sorex sinalis*	NC_037174.1
*Sorex*	Tibetan Shrew	*Sorex thibetanus*	NC_064993.1
*Sorex*	Tundra Shrew	*Sorex tundrensis*	NC_025327.1
*Sorex*	Long-Clawed Shrew	*Sorex unguiculatus*	AB061527.1
*Soriculus*	Himalayan Shrew	*Soriculus nigrescens*	NC_052688.1
*Suncus*	Asian House Shrew	*Suncus murinus*	PX591023.1

## Data Availability

The raw data have been deposited in the NCBI SRA database under project ID PRJNA1397560 with accession numbers SRR36667617 (CH, 10 Gb), SRR36667616 (CL, 10 Gb), SRR36667615 (CS1, 10 Gb), and SRR38325269 (CS2, 40 Gb).

## Supplementary Materials

The following supporting information can be downloaded at https://www.mdpi.com/article/10.3390/ani16101471/s1, Supplementary Table S1: Overview of the complete mitochondrial genome of *Chimarrogale himalayica*. Supplementary Table S2: Overview of the complete mitochondrial genome of *Chimarrogale leander*. Supplementary Table S3: Overview of the complete mitochondrial genome of *Chimarrogale styani*. Supplementary Table S4: Nucleotide composition and skewness of PCGs of three *Chimarrogale* species.
